# Fruit and vegetables intake among elderly Iranians: a theory-based interventional study using the five-a-day program

**DOI:** 10.1186/1475-2891-10-123

**Published:** 2011-11-14

**Authors:** Leili Salehi, Kazem Mohammad, Ali Montazeri

**Affiliations:** 1 Tehran University of Medical Sciences, Center for Community Based Participatory Research (CBPR), Tehran, Iran; 2 Alborz University of Medical Sciences, Karaj, Iran; 3 Department of Epidemiology and Statistics, School of Public Health, Tehran University of Medical Sciences, Tehran, Iran; 4 Mental Health Research Group, Mother and Child Health Research Centre, Iranian Institute for Health Sciences Research, ACECR, Tehran, Iran

## Abstract

**Background:**

The benefit of FV intake in old age is well documented. However, there is evidence that old people do not consume enough FV. The purpose of this study was to evaluate the effectiveness of a tailored nutrition intervention that aimed to increase the FV intake among elderly Iranians aged 60 and over.

**Methods:**

This quasi-experimental study was performed among a community-based sample of elderly in Tehran, Iran in year 2008 to 2009. Data were collected at baseline and 4 weeks follow-up. At baseline face-to-face interviews were conducted using a structured questionnaire including items on demographic information, stages of change, self-efficacy, decisional balance, daily servings of FV intake. Follow-up data were collected after implementing the intervention.

**Results:**

In all 400 elderly were entered into the study (200 individuals in intervention group and 200 in control group). The mean age of participants was 64.06 ± 4.48 years and overall two-third of participants were female. At baseline total FV intake was not differed between two groups but it was significantly increased in the intervention group at posttest assessment (mean serving/day in intervention group 3.08 ± 1.35 vs. 1.79 ± 1.08 in control group; P = 0.001). Further analysis also indicated that elderly in intervention group had higher FV intake, perceived benefits and self-efficacy, and lower perceived barriers. Compared with control group, greater proportions of elderly in intervention group moved from pre-contemplation to contemplation/preparation and action/maintenance stages (P < 0.0001), and from contemplation/preparation to action/maintenance stages (P = 0.004) from pretest to posttest assessments.

**Conclusion:**

This study suggests that the Transtheoretical Model is a useful model that can be applied to dietary behavior change, more specifically FV consumption among elderly population in Iran and perhaps elsewhere with similar conditions.

## Background

Adequate FV intakes could decrease risk of various chronic diseases such as cancers, cardiovascular disease, diabetes, and stroke [1-6]. The World Health Organization (WHO) dietary guideline recommends the minimum 5-a-day consumption of FV [7]. Individuals have not, as yet, adopted the minimum recommendations to consume five servings of FV per day in spite of all the benefits of FV on improving health and reducing the economic burdens of chronic disease. Based on data obtained from FV intakes in 21 countries (mainly developing countries) only in three countries FV intake met the minimum WHO recommended consumption [8].

The benefit of FV intake in old age is well documented [9-11]. However, there is evidence that old people do not consume enough FV. For instance a study from Canada showed that only about 47% of elderly consume recommend amount of FV [12]. Data from a study of 400 elderly in Iran showed that FV consumption among participations was low. Overall the mean serving of FV consumption eaten per day for the elderly was 1.76 (SD = 1.15) [13]. Another cross sectional study showed that the prevalence of daily FV intake of 5 or more serving was 37% and the mean daily FV consumption separately was 1.86 ± 0.68 and 2.74 ± 0.83 respectively [14].

Such observations from Iran and elsewhere indicate that there is urgent need for health promotion programs in order to increase FV consumption among elderly population. But, the debate about effectiveness of these programs still remains [15]. It is argued that these programs, at least, should be theory driven if one expects any appropriate changes in dietary behaviors [16]. The Transtheoretical Model (TTM) of behavior change is one of the most popular models for studying behavioral change in health education/promotion programs. This model assumes that health behavior change involves progress through six stages: pre-contemplation (unaware of a problem and/or not intending to change), contemplation (considering a change and thinking about it), preparation (intending to take action in very near future), action (initiating a new behavior), maintenance (in which people strive to prevent relapse), and termination (in which individuals show complete self-efficacy) [17].

Several studies showed that dietary interventions based on stages of change model are effective in increasing FV intake [18-20]. Only a few studies however have been conducted using the TTM for elderly population [21,22]. The purpose of this study was to evaluate the effectiveness of a tailored nutrition intervention that aimed to increase the FV intake among elderly Iranians. According to the report by Iranian Ministry of Health the proportion of elderly (≥ 65) in Iran accounts for 5.4 percent and it is estimated that this rate will be rise to 10.5 percent by 2025 [23]. To our best knowledge this is the first paper from Iran that reports on the topic.

## Methods

### Design and data collection

This quasi-experimental study was performed among a community-based sample of elderly people aged 60 and over in Tehran, Iran in year 2008 to 2009. Data were collected at baseline and 4 weeks follow-up. At baseline face-to-face interviews were conducted with the whole sample using a structured questionnaire including items on demographic information, stages of change, self-efficacy, decisional balance, daily servings of FV intake. Four weeks follow-up data were collected after implementing the intervention.

### Participants

Of 30 existing elderly centers in Tehran, 10 centers were randomly selected through multistage sampling to represent centers from all 5 main areas in Tehran (2 centers from each area: south, north, east, west and city center). Within the 10 selected centers the membership list for each center was asked and relative to density a systematic sampling method was applied. The selected participants contacted and asked if they were willing to participate in the study. Participants were also informed about the study and the number of required meetings during the study. Participants were then randomized to the intervention or the control groups. A health professional not connected to the study carried out the randomization. People in both groups received a four-cession program.

### Measures

Several instruments were used to collect data:

#### 1. Demographic and anthropometrics Questionnaire

This comprised three sections covering demographic and anthropometrics data including information on age, sex, education, income, marital status, health status (having chronic disease or not) and body mass index (BMI). Chronic disease was indicated by asking each individual to respond to the following question: 'Do you have any long-standing disease?' Anyone who responded positively then was asked to name the disease. Weight was measured using the same digital scales [SECA, Calibrated in Iran] while the participants were minimally clothed and not wearing shoes. Height was measured by a tape measure while the respondents were standing and not wearing shoes and the shoulders were in a normal position. BMI was calculated and expressed in kg/m^2^, and economic status was measured using the asset-based approach developed by Ferguson and colleagues [24] and used in previous cross-national studies of economic status and health in developing countries [25]. According to this scale, 0-3 assets were considered low, 4-6 assets were considered intermediate and 8 or more assets were considered high economic status. The items considered as assets were: television, refrigerator, washing mashing, microwave oven, dish-washer, computer, electrical sweeper, automobile and phone.

#### 2. Stages of change questionnaire regarding FV consumption behavior

This part of questionnaire adapted from the literature [26] and was consisted of five statements by which the participants were categorized into different stages of change: pre-contemplation, contemplation, preparation, action and maintenance. In fact the participants were asked to respond to one question choosing the statement that best described their status. Choices for the response were: (1) I am not currently consuming five servings of FV a day and I am not thinking of doing so in the upcoming six months, (2) I am not currently consuming five servings of FV a day but I plan to do so within the next six months. (3) I am not currently consuming five servings of FV a day but I plan to do so within the next month (4) I am currently consuming five servings of FV a day but I have been doing so for less than six months (5) I am currently consuming five servings of FV a day but I have been doing that for more than six months.

#### 3. Self-efficacy

Self-efficacy was assessed to measure confidence in one's ability to persist with FV consumption in various situations. It was assessed using a five item questionnaire developed by Ma et al. [26] and participants were asked to respond to these items: 'I can keep fruits and vegetables at hand/readily available'; 'When I have the chance to choose, I can eat the recommended number of servings of fruits and vegetables'; 'I can shop for a variety of fruits and vegetables'; 'I can make time to eat fruits and vegetables'; 'When I eat at home, I can eat more fruits and vegetables'. Each item is rated on a 5-point scale (from not at all confident = 0 to very confident about recommended FV consumption = 5). The total score ranged from 5 to 25 with higher scores indicating a greater degree of self-efficacy.

#### 4. *Perceived benefits and barriers regarding FV consumption*

This part was generated from previous studies and focus group discussions with convenient sample of elderly individuals. Participants were asked about their perception regarding amount of FV intake. The perceived benefit consisted of 15 items and each item is rated on a 5-point scale ranging from 'strongly agree' to 'strongly disagree'. The perceived barrier consisted of 11 items and each item is also rated on a 5-point scale ranging from 'strongly agree' to 'strongly disagree'. The total score for the perceived benefits ranged from 15 to 75 and for perceived barriers from 11 to 55 (Table 1).

**Table 1:** Perceived benefits and barriers questionnaire

	Strongly agree	Agree	Neither agree nor disagree	Disagree	Strongly disagree
**Perceived Benefits**					

I could find any types of FV in my local stores					

It is better to get all nutrients from FV than taking supplements					

FV decrease the risk of chronic diseases					

FV make our diet diverse					

Eating FV is a good way for treating chronic diseases					

Eating FV would help me to be less aggressive					

Eating FV treats constipation					

Eating FV would help me to maintain my weight					

FV consumption are recommended by physicians					

Eating FV cheering my family members					

Eating FV is common in my culture					

Eating FV would keep me of sickness					

Eating FV would help me to live longer					

I feel that if I eat more FV, I will be more healthy					

By eating FV, I feel better					

**Perceived Barriers**					

Providing FV is expensive					

I did not used to eat FV since childhood					

Eating FV leads to overeating					

Media advertisements are not about eating FV					

Eating more FV is not recommended in my culture					

My family members do not like consumption of FV					

Eating more FV is difficult for me					

I have health problems with eating FV (e.g. flatus)					

I have limitations to provide FV in my meal					

I do not like taste of FV					

I do not have time to provide FV					

Please indicate whether you agree or disagree with the following items when you are deciding on consuming or not to consume FV. Check the best response.

#### 5. Daily FV consumption

The section comprised two parts as follows.

##### 5.1. Food frequency questionnaire

This was consisted of two main questions related to fruits and vegetables (38 items in all) available in Tehran's markets. Response categories were: never, 1-2 times per week, 3-4 times per week, 5-6 times per week, and every day. Accordingly the respondents were asked to indicate the amount of intake.

##### 5.2. A 24 hour recall

participants were asked to estimate their daily servings of FV at breakfast, lunch, dinner, and between meals as snacks or deserts in accordance with a nutrition guideline cards. The nutrition guideline card categorized one serving of vegetables into one of three following groups: (1) one cup of raw green leafy vegetables such as spinach or salad; (2) one-half cup of other vegetables cooked or chopped raw, such as tomatoes, carrots, pumpkin, corn, Chinese Cabbage, beans, or onions; and (3) one half cup of vegetable juice. The nutrition guideline categorized one serving of fruit into one of three groups: (1) one medium size fruit such as an apple, banana, or orange; (2) one half cup of cooked, chopped, or canned fruit; and (3) one-half cup of fruit cup of fruit juice, not artificially flavored.

We calculated the daily serving FV consumption for each individual according to information provided from the above-mentioned measures. This included of calculation of weekly consumption of FV based on frequency and portion of each item in the food frequency questionnaire. Then we compared total score of daily FV consumption between two groups.

### Intervention

Both those randomized to the control group and those randomized to the intervention group received four weekly sessions. The control group sessions focused on general health education and did not include content related to the health benefits of fruits and vegetables, while the intervention group sessions were focused on increasing fruit and vegetable intake.

After randomization, those randomized to intervention group were further divided by stages of change and the sessions were then tailored to that stages of change and techniques (processes) associated with the stages of change. The goal of the intervention was to increase participants' consumption of FV to 5 servings per day. The intervention was composed of four consecutive sessions (one meeting per week). Each session was around 90 minutes in length and included a 40-minute power point presentation, 30 minutes discussion, 10 minutes questions and answers, and 10 minutes reception with FV.

(I) The first session was introductory.

(II) During the second session, the stages of change for FV intake was assessed in participants in order to deliver the appropriate intervention. Based on each individual's status at least one tailored technique (according to the Transtheoretical Model) was used:

(II-a) Participants in pre-contemplation stage completed session that incorporated conscious raising (raising awareness about unhealthy dietary behavior); dramatic relief (react emotionally to warnings about unhealthy dietary behavior. The topics included personal recommendation regarding losing a loved one due to a chronic disease and discussion of nutritional habits associated with this chronic disease; emotional arousal (which is a certain technique that produce increased emotional experiences that can encourage people towards an action. In fact, this process of behavioral change was used to help the participants understand the relationship between lower consumption of FV and increased risk for chronic diseases. The topics included the statistics about the prevalence of the previously mentioned chronic diseases in world and in Iran, the scientific studies relating the protective effect of FV against each of these diseases); and environmental reevaluation (assessing the impact of one's dietary behavior on family members and others).

(II-b) Participants in contemplation/preparation stage completed session that incorporated self-evaluation (which is an assessment of one's self-image with and without a particular unhealthy habit. The researcher asked the participants who were now consuming 5 servings of FV per day to compare their lifestyle and diet before and after increasing their intake of FV to five or more servings per day); and self-liberation (which is the belief that one can change and have the commitment to act on that belief. Participants were asked to make a plan and set a goal and be committed to that goal).

(II-c) Participants in the action/maintenance stage completed session that incorporated helping relationship (which is defined as having a caring, trusted, and accepted person who can give the support and the counseling for the healthy behavior change), stimulus control (removing or countering stimuli that elicit problem behavior), reinforcement management (rewarding oneself or being rewarded by others for making dietary change).

(III) During the third session, the content of the second session was reinforced.

(IV) The fourth session was planned to help participants anticipate and overcome barriers, increase self-assurance and self-efficacy in addition to improve skills in obtaining and arranging FV.

### Statistical Analysis

The characteristics of participants in two groups were compared using analysis of variance and x^2 ^tests as appropriate. Responses to the interventions were assessed by calculating changes in fruit and vegetable intake from baseline to 4 weeks, with positive values indicating an increase in consumption at follow-up assessment. Similar analysis was performed for assessment of stages of change (posttest data vs. pre-contemplation, contemplation/preparation, and action/maintenance at baseline). The data were analyzed on an intention to treat basis including all 400 participants. Analysis of covariance (ANCOVA) was used, controlling for variables previously shown to be related to FV consumption, namely, age, education, marital status, income, chronic disease and BMI. All analyses were conducted using SPSS 16.0. Alpha level of .05 was used for all statistical tests.

### Outcome measures

Two main outcomes of the current study were changes in FV intake and to examine the stage transitions.

### Ethics

Ethics committee of Tehran University of Medical Sciences approved the study. All participants singed a consent form.

## Results

### The study samples

In all 400 elderly were entered into the study (200 individuals in intervention group and 200 in control group). The two groups did not differ in terms of demographic characteristics. The mean age of participants was 64.0 6 (SD = 4.48) years and overall two-third of participants were female (n = 298, 74.5%). The characteristics of participants in the two groups are shown in Table 2.

**Table 2:** The characteristics of the study sample

	Total	Intervention group (n = 200)	Control group (n = 200)	
	**No. (%)**	**No. (%)**	**No. (%)**	**P**

**Age**				0.55

60-64	255 (63.8)	120 (65.0)	125 (62.5)	

65-69	87 (21.7)	34 (17.0)	53 (26.5)	

70-74	47 (11.7)	30 (15.0)	17 (8.5)	

≥ 75	11 (2.8)	6 (3.0)	5 (2.5)	

Mean (SD)	64.06 (4.48)	63.93 (5.08)	64.2 (3.8)	

**Gender**				

Female	298 (74.5)	144 (72.0)	154 (77.0)	0.81

Male	102 (25.5)	56 (28.0)	46 (23.0)	

**Education**				0.08

Illiterate	165 (41.2)	82 (41.0)	83 (41.5)	

Primary	143 (35.8)	82 (41.0)	61 (30.5)	

Junior Secondary	64 (17.0)	23 (11.5)	41 (20.5)	

Senior Secondary & above	28 (7.0)	13 (6.5)	15 (7.5)	

**Marital status**				

Married	230 (55.0)	120 (60.0)	110 (55.0)	0.45

Never married/Divorced/widow	170 (45.0)	80 (40.0)	90 (45.0)	

**Income**				0.03

Low (0-3assets)	306 (76.5)	165 (82.5)	141 (70.5)	

Moderate (4-6 asset)	65 (16.2)	22 (11.0)	43 (21.5)	

High (8 or more assets)	29 (7.3)	13 (6.5)	16 (8.0)	

**Employment status**				0.45

Employed	54 (13.5)	31 (15.5)	23 (21.5)	

Housewife	283 (70.8)	133 (66.5)	150 (70.0)	

Retired	63 (15.7)	36 (18.0)	27 (13.5)	

**BMI**				0.79

< 25	106 (26.5)	56 (28.0)	50 (25.0)	

25-29	192 (48)	94 (47.0)	98 (49.0)	

≥ 30	103 (25.5)	50 (25.0)	52 (26.0)	

### Change in FV intake

At baseline total FV intake was not differed between two groups (intervention and control groups) but it was significantly increased in the intervention group at posttest assessment (mean serving/day in intervention group 3.08 ± 1.35 vs. 1.79 ± 1.08 in control group; P = 0.001). The detailed results are shown in Table 3 and Table 4. Pearson correlation also showed significant correlation between stages of change and benefits, barriers, self-efficacy and FV intake (Table 5). Further analysis of the data performing the analysis of variance adjusting for covariate also indicated that there were significant differences between intervention and control groups Elderly in intervention group had higher FV intake, perceived benefits and self-efficacy, and lower perceived barriers. The results are presented in (Table 6).

**Table 3:** Comparison of perceived benefits, perceived barriers, self-efficacy and FV consumption between two groups

	Before			After		
	**Intervention group**	**Control group**		**Intervention group**	**Control group**	

	**Mean (SD)**	**Mean (SD)**	**P**	**Mean (SD)**	**Mean (SD)**	**P**

**Perceived Benefits**	54.91 (9.29)	56.62 (8.34)	0.21	64.34 (9.11)	57.01 (8.47)	< 0.001

**Perceived Barriers**	34.23 (8.04)	35.79 (8.29)	0.50	27.32 (8.09)	35.89 (8.72)	< 0.001

**Self-efficacy**	13.59 (6.41)	12.72 (6.02)	0.22	19.23 (5.72)	12.79 (6.04)	< 0.001

**FV Consumption**	1.78 (1.21)	1.75 (1.09)	0.17	3.08 (1.36)	1.79 (1.08)	< 0.001

**Table 4:** Comparison of perceived benefits, perceived barriers, self-efficacy and FV consumption within groups

	Intervention group			Control group		
	**Before**	**After**		**Before**	**After**	

	**Mean (SD)**	**Mean (SD)**	**P**	**Mean (SD)**	**Mean (SD)**	**P**

**Perceived Benefits**	54.91 (9.29)	64.35 (9.11)	< 0.001	56.62 (8.34)	57.01 (8.47)	< 0.001

**Perceived Barriers**	34.23 (8.04)	27.33 (8.09)	< 0.001	35.79 (8.29)	35.89 (8.72)	0.262

**Self-efficacy**	13.59 (6.41)	19.23 (5.72)	< 0.001	12.72 (6.02)	12.79 (6.04)	0.007

**FV Consumption**	1.78 (1.21)	3.08 (1.36)	< 0.001	1.75 (1.09)	1.79 (1.08)	0.006

**Table 5:** Correlation between stage of change, benefits, barriers, self-efficacy and FV consumption*

	Stages of change	Benefits	Barriers	Self-efficacy	FV consumption
**Stages of change**	1	0.213**	-0.181**	0.181**	0.448**

**Benefits**	0.371**	1	-0.033	0.096	0.237**

**Barriers**	-0.403**	-0.217**	1	-0.173**	-0.296**

**Self-efficacy**	0.437**	0.303**	-0.375**	1	0.337**

**FV consumption**	0.636**	0.355**	-0.402**	-0.485**	1

* Figures above triangle relate to before intervention and figures below triangle relate to after intervention

** Correlation is significant at the 0.01 levels.

**Table 6:** Analysis of covariance of perceived benefit, perceived barrier, self-efficacy, and FV consumption

Source of variance	Type III sum of square	**df**.	Mean square	F statistic	P
**Perceived Benefit**					

Arm	6846.086	1	6846.086	179.074	< 0.0001

Pretest	15388.664	1	15388.664	402.523	< 0.0001

Age	14.127	1	14.127	0.37	0.544

Education	11.286	1	11.286	0.295	0.587

Marital status	77.527	1	77.527	2.028	0.155

Income	65.055	1	65.055	1.702	0.193

Chronic disease	50.452	1	50.452	1.32	0.251

BMI	41.750	1	41.750	1.092	0.297

Error	14948.144	391	38.231		

**Perceived Barrier**					

Arm	5423.038	1	5423.038	174.115	< 0.0001

Pretest	13877.096	1	13877.096	455.545	< 0.0001

Age	38.961	1	38.961	1.251	0.264

Education	80.167	1	80.167	2.574	0.109

Marital status	19.014	1	19.014	0.61	0.435

Income	50.268	1	50.268	1.614	0.205

Chronic disease	2.029	1	2.029	0.065	0.799

BMI	0.172	1	0.172	0.006	0.941

Error	12178.223	391	31.146		

**Self-efficacy**					

Arm	3252.374	1	3252.374	475.144	< 0.0001

Pretest	9563.134	1	9563.134	1.397	< 0.0001

Age	23.900	1	23.900	3.492	0.062

Education	5.188	1	5.188	0.758	0.385

Marital status	1.006	1	1.006	0.147	0.703

Income	30.368	1	30.368	4.436	0.036

Chronic disease	1.022	1	1.022	0.149	0.699

BMI	0.282	1	0.282	0.041	0.839

Error	2676.407	391	6.845		

**FV consumption**					

Arm	156.226	1	156.226	294.929	< 0.0001

Pretest	218.617	1	218.617	412.712	< 0.0001

Age	2.400	1	2.400	4.530	0.034

Education	0.017	1	0.017	0.032	0.858

Marital status	1.301	1	1.301	2.456	0.118

Income	0.055	1	0.055	0.105	0.746

Chronic disease	0.198	1	0.198	0.374	0.541

BMI	0.062	1	0.062	0.118	0.732

Error	207.116	391	0.530		

### Change in stage transition

There was no difference at baseline in distribution of the stages of change between the two groups. Larger number of participants fell into the pre-contemplation stage than the other stages. Compared with control group, greater proportions of elderly in intervention group moved from pre-contemplation to contemplation/preparation and action/maintenance stages (χ^2 ^= 233.7, P < 0.0001), and from contemplation/preparation to action/maintenance stages (χ^2 ^= 8.1, P = 0.004) from pretest to posttest measurements (Table 7).

**Table 7:** Chi-square analysis of between groups differences in posttest stages of change by pretest stages of change*

	Post test stages of change				
	
	*PC*	*C/PR*	*A/M*	*χ^2 ^(df)*	*P*
**Pretest stages of change**					

*PC*					

Intervention (n = 140)	5	117	18		

Control (n = 143)	135	8	0		

				233.7 (2)	< 0.0001

*C/PR*					

Intervention (n = 53)	0	45	8		

Control (n = 50)	0	50	0		

				8.1 (1)	0.004

A/M**					

Intervention (n = 7)	0	0	7		

Control (n = 7)	0	0	7		

PC = Pre-contemplation, C/PR = Contemplation/Preparation, A/M = Action/maintenance

* The format of table was adapted from [28].

** Note that it is impossible to progress from action/maintenance.

## Discussion

This study indicated the efficacy of a TTM-based intervention for increasing fruit and vegetable consumption in elderly. The findings also confirmed that a theory driven program could have effect on stages of change in elderly in order to improve their lifestyle and health behavior.

We found an average increase of 1.29 daily servings of fruits and vegetables in the intervention group. The results from current study were very similar to those reported by other investigators. For instance, an interventional study that applied the TTM to promote FV consumption showed that FV intake in elderly increased from 0.5 to 1.0 serving a day [22]. Another study reported a significant increase in FV consumption; 1.49 serving/day increase when using a theory based intervention [27]. Di Noia et al. conducted the TTM based study with urban African-American adolescents to determine whether the delivery of stage-tailored change process would promote movement through successive stages and effect positive changes in FV consumption, pros, cons and self efficacy and found that the intervention group had greater increase in the perceived pros of eating FV and increase of 0.9 daily serving of FV compared with the control group [28]. Similarly our findings demonstrated that the intervention had positive effect on perceived benefits and barriers, and self-efficacy. We believe the strength of the current study was due to the fact that our intervention not only was drawn from the TTM stages of change, but also it included examination of perceived barriers and self-efficacy.

Overall most interventional studies based on the TTM model showed that the interventions have produced positive influence on FV intakes. However, the program developed by Amanda Park indicated that stage-tailored nutrition education produced positive shift in several indicators and mediators of vegetables but not for fruits intake [29]. Our study assessed both fruits and vegetables together. Hence it is better to assess the two items apart in future studies as a match-mismatch test. A study was conducted to test the transtheoretical model applied to fruit intake and failed to support the superiority of stage-matching compared with stage-mismatching [30].

## Limitations

Given that all our respondents were members of elderly centers, the findings of this study might not be generalized to all elderly Tehran residents. These elderly might differ from others in Tehran in terms of socioeconomic status, family cohesiveness, social support and availability and access to FV. In addition, it should be noted that our findings on FV intake were based on self-reported information and it might be associated with measurement errors.

## Conclusion

This study demonstrated that the TTM is a useful model that can be applied to dietary behavior change, more specifically FV consumption among elderly populations.

## Competing interests

The authors declare that they have no competing interests.

## Authors' contributions

LS was the main investigator, analyzed the data and involved in drafting the manuscript. KM contributed to the study design and statistical analysis, and supervised the study. AM contributed to statistical analysis, edited the paper and provided the final version. All authors read and approved the final manuscript.
